# Preoperative Stereotactic Body Radiotherapy to Portal Vein Tumour Thrombus in Hepatocellular Carcinoma: Clinical and Pathological Analysis

**DOI:** 10.1038/s41598-020-60871-0

**Published:** 2020-03-05

**Authors:** Noriko Kishi, Naoyuki Kanayama, Takero Hirata, Shingo Ohira, Kentaro Wada, Yoshifumi Kawaguchi, Koji Konishi, Shigenori Nagata, Shin-ichi Nakatsuka, Shigeru Marubashi, Akira Tomokuni, Hiroshi Wada, Shogo Kobayashi, Yasuhiko Tomita, Teruki Teshima

**Affiliations:** 1 Department of Radiation Oncology, Osaka International Cancer Institute, 3-1-69 Otemae, Chuo-ku Osaka, 541-8567 Japan; 2 Department of Diagnostic Pathology and Cytology, Osaka International Cancer Institute, Osaka, Japan; 3 Department of Surgery, Osaka International Cancer Institute, Osaka, Japan; 4 Department of Surgery, Osaka University Graduate School of Medicine, Suita Osaka, Japan

**Keywords:** Hepatocellular carcinoma, Surgical oncology

## Abstract

The prognosis of hepatocellular carcinoma (HCC) with portal vein tumour thrombus (PVTT) is poor. We conducted a prospective study to evaluate the efficacy and safety of tri-modality therapy, including preoperative stereotactic body radiotherapy (SBRT) and surgery, followed by hepatic arterial infusion chemotherapy (HAIC) in HCC patients with PVTT. In this report, we investigated the pathology of the irradiated PVTT specimen in resected cases and SBRT-related acute toxicity. A total of 8 HCC patients with PVTT received preoperative SBRT targeting the PVTT at a dose of 48 Gy in 4 fractions at our institute from 2012 to 2016. Of the eight patients, six underwent surgery, while the remaining two did not because of disease progression. At the pathological examination, all patients’ irradiated PVTT specimens showed necrotic tissue, and three of six patients showed complete pathological response. Two patients showed 30% necrosis with high degeneration and one patient, with 30% necrosis without degeneration, was the only recurrent case found during the follow-up period (median: 22.5, range: 5.9–49.6 months). No SBRT-related acute toxicity worse than grade 2 was observed from SBRT to surgery. In conclusion, the preoperative SBRT for HCC was pathologically effective and the acute toxicities were tolerable.

## Introduction

Hepatocellular carcinoma (HCC) is the fifth most common cancer worldwide and ranks third in Asia^1^. The treatment strategy for HCC patients who meet the Milan criteria (single lesion ≤5 cm, or ≤3 lesions ≤3 cm; no extrahepatic lesions; no macrovascular invasions) is liver transplantation. In resectable cases with preserved liver function, hepatectomy is performed. For patients who are not eligible for curative surgery, local therapies including radiofrequency ablation (RFA), transcatheter arterial chemo-embolisation (TACE) and radiotherapy are considered.

Curative surgery in patients with macrovascular invasion is controversial. The prognosis of HCC with portal vein tumour thrombus (PVTT) is poor and the median survival time is 2.7–4.0 months^2,3^. Hepatectomy confers a median survival of 8.6–11 months and a perioperative mortality rate of 5.9%^4–6^. Patients with HCC and PVTT are often ineligible for liver transplantation, and the outcome is poor^7–9^. TACE is generally contraindicated because of the size, location, and the risk of liver failure, though improved outcomes have been reported^10,11^. Oral multi-kinase inhibitors, such as sorafenib and regorafenib are systemic treatment options for HCC patients with PVTT, which improves overall survival (OS) for 2 months only^12–14^; there is no other evidence-based monotherapy for improving survival. Combination therapy, such as neoadjuvant TACE plus transplantation is considered to be promising^15^. Preoperative intervention decreases tumour cell viability, shrinks the PVTT, and may improve the resection rate. Therefore, local therapies as a bridging or down-staging therapy have come to play an important role in HCC patients with PVTT.

The use of preoperative three-dimensional conformal radiotherapy (3D-CRT) for HCC with PVTT was first reported at our institute^16^. Kamiyama, *et al*. reported that preoperative 3D-CRT significantly improved survival outcomes after hepatic resection^17^. Using conventional fractionation and 3D-CRT, 4–6 weeks are needed to deliver 50–60 Gy to the target volume; in addition, wide set-up and internal margins are needed to account for respiration-induced liver motion. The prolonged treatment periods may induce progression of radioresistant tumour clones and the large irradiated fields may increase radiation toxicity. Stereotactic body radiotherapy (SBRT) shortens overall treatment time, delivers highly conformal ablative radiotherapy to small irradiated volumes, and accomplishes low irradiated doses to organs-at-risk (OARs). The case we previously reported indicated that preoperative SBRT and hepatic surgery can be a local down-staging treatment option^18^. We conducted a prospective study to evaluate the efficacy and safety of tri-modality therapy including preoperative SBRT and surgery, followed by hepatic arterial infusion chemotherapy (HAIC) in HCC patients with PVTT. In this report, we focused on the pathology of the irradiated PVTT specimen in resected cases and SBRT-related acute toxicity in HCC patients who received preoperative SBRT targeting PVTT.

## Methods

### Eligibility criteria

The aim of the prospective study sub-analysis was to investigate the pathological response of the irradiated PVTT specimen and SBRT-related acute toxicities in HCC patients who underwent preoperative SBRT at our institute. The eligibility criteria for preoperative SBRT were as follows: 1) lesions confined to the liver hemi-lobe or estimated remnant liver fulfilling the Milan criteria; 2) HCC with PVTT in the second branch (Vp2), the first branch (Vp3), or the trunk of the portal vein, or in a branch on the opposite side (Vp4), according to the general rules for the clinical and pathological study of primary liver cancer (The 6^th^ edition)^19^; 3) no distant metastasis; and 4) received treatment between July 2012 and April 2016. The exclusion criteria were as follows: 1) medically inoperable major organ dysfunction (including bone marrow, heart, liver, kidney, and lung dysfunction); 2) Eastern Cooperative Oncology Group Performance Status 2 or greater; 3) did not undergo surgery after SBRT for any reason; and 4) did not provide written consent prior to SBRT.

### Patient characteristics

We investigated tumour factors (number, size, segment, and serum alpha-fetoprotein), liver function (Child-Pugh score, indocyanine green retention at 15 minutes, and des-γ-carboxy prothrombin [DCP]), and the aetiology of liver disease (hepatitis viral infection, history of alcohol abuse, and body mass index). All tumours were staged using various clinical systems (Okuda staging, Barcelona Clinic Liver Cancer staging, Cancer of the Liver Italian Program score)^20^ and staged as T4N0M0 according to the TNM classification of malignant tumours, Union for International Cancer Control (UICC) 8^th^ edition. This study was performed in accordance with the principles of the Declaration of Helsinki (1975, as revised in 2013). Informed consent was obtained from all individual participants included in the study, and the Institutional Review Board of the Osaka International Cancer Institute approved this study in July 2016. Our IRB numbers are 1303075150 and 1606299040.

### Treatment protocol

Contrast-enhanced computed tomography (CT) scans performed before and after SBRT were evaluated by a cancer board including surgeons, medical oncologists, diagnostic radiologists, and radiation oncologists for tumour resectability and disease progression.

Patients were instructed to fast for 3 hours prior to the simulation to avoid undue displacement of abdominal organs owing to stomach bulk; patients were immobilised using a vacuum pillow (Vac-Lok^TM^) in a supine position with the arms raised. Under abdominal compression, free-breathing four-dimensional CT (4D-CT) and breath-holding CT (slight expiration and inspiration) scans were performed using the LightSpeed16 scanner (GE Medical Systems, Waukesha, WI). The parameters for CT acquisitions were: 2.5 mm slice thickness, 512 × 512 matrix, and a 50 cm field of view. Using the Advantage Sim workstation (GE Medical Systems), a phase-based method was used to generate 10 respiratory phase 4D-CT images.

The shadow of the diaphragm or the shadow of lipiodol from a prior TACE treatment was used as a surrogate marker for respiratory motion. If neither can be used, two fiducial markers (Visicoil, Core Oncology, Santa Barbara, CA) were placed near the target lesion under ultrasound guidance to visualize the tumour position and respiratory motion as a surrogate marker (respiratory-gated SBRT). For tumours with <10 mm respiratory motion, free-breathing volumetric modulated arc therapy (VMAT) with abdominal compression using a plastic sheet (respiratory-suppressed SBRT) was performed. If abdominal compression was not enough to suppress the respiratory motion <10 mm, infrared (IR)-marker based respiratory-gated radiotherapy with multiple 6–7 non-coplanar photon was performed.

Gross tumour volume (GTV) was contoured on the 0% and 50% CTs to encompass the entire respiratory motion for respiratory-suppressed SBRT, while the GTV was contoured on the 40%, 50% and 60% CTs for respiratory-gated SBRT. Internal clinical target volume (iCTV) was defined as the fusion of clinical target volumes in multiple 4D-CT phases; a 0–5 mm isotropic margin was added to the sum of GTVs to create the iCTV. A 5–8 mm margin was added to the iCTV to generate the planning target volume (PTV). During contouring, an imaginary cutting line, estimated remnant liver volume, and the PVTT-target volume were delineated after discussion with the surgeons. OARs were contoured on the average CT (Ave-CT) generated from the 10-phase 4D-CT image. Treatment plans were generated using a treatment planning system (Eclipse, Varian Medical Systems), and doses were calculated using the Anisotropic Analytical Algorithm (AAA). Treatment was delivered with a D95 prescription of 48 Gy in 4 fractions for 4 consecutive days except on Saturdays and Sundays. Dose constraints were defined for the spinal cord (V20 Gy <1 cc, V15 Gy <10 cc), stomach (Dmax <27.5 Gy, V20 Gy <5 cc), duodenum (Dmax <16 Gy) and bowels (Dmax <16 Gy)^21^. The beams were arranged to traverse the liver portions designated for resection, while the estimated remnant liver would receive minimal doses. Treatment was delivered using a linear accelerator (Clinac 23EX, Varian Medical Systems) with patients immobilised in the treatment position, which was identical to that of the CT simulations. Patients underwent hepatic lobectomy within 2 weeks after SBRT. Therapeutic effect was assessed by histopathological analysis of resected Victoria blue and haematoxylin-eosin [VB-HE] -stained PVTT specimens. After recovery from surgery, an implantable reservoir was placed prior to adjuvant chemotherapy. Low-dose intra-arterial infusions of 5-fluorouracil (1250 mg/m^2^) and cisplatin (50 mg/m^2^) were administered for 5 days/week for 3 cycles, with 2 weeks on and 2 weeks off.

### Endpoints and statistical analysis

We evaluated the pathological response of the irradiated PVTT specimen, the percentage of necrosis, presence/absence of degeneration, and the association between response to radiotherapy and tumour size, differentiation, or the period from SBRT to surgery. SBRT-related acute toxicities were defined as adverse events (AEs) occurring from the day of initiation of SBRT to the day of surgery, which were graded using the National Cancer Institute Common Terminology Criteria for Adverse Events, version 4.0.

Gastrointestinal haemorrhage, ulcers, and strictures, and radiation-induced liver dysfunction (RILD) occurring during the follow-up period were recorded. RILD was classified into two types, namely, classic (involving anicteric hepatomegaly, ascites, and elevated alkaline phosphatase) and non-classic (elevated liver transaminases or a decline in liver function without the classic-type features)^21^. Furthermore, dose-volume data of the PTV, normal liver (whole liver excluding the PTV), the estimated remnant liver, and OARs (spinal cord, stomach, duodenum, and bowel) were also collected.

Intrahepatic progression was defined as tumour recurrence in the remnant liver. Intrahepatic recurrence-free survival (IHRFS) was defined as the interval between the day of surgery and the day of intrahepatic progression and was censored on the day on which intrahepatic recurrence-free survival was verified by abdominal CT or ultrasonography. Extrahepatic recurrence-free survival (EHRFS) was defined as the interval between the day of surgery and the date of any recurrence outside the liver. Kaplan–Meier analysis and the log-rank test were used to calculate IHRFS and EHRFS.

Statistical analysis was performed using the R Version 3.5.1 (The R Foundation for Statistical Computing) software package.

## Results

### Patient characteristics

Among the eight patients who received preoperative SBRT targeting the PVTT, two patients were excluded because they did not undergo surgery; one developed intrahepatic metastasis, and the remaining one demonstrated primary tumour progression. Therefore, we investigated the six eligible postoperative patients. All were male with a median age of 72.5 years (range: 61−78 years); four cases had initial tumours and two had recurrent tumours. TACE and subsegmentectomy+TACE were administered in one patient each. Respiratory-gated multiple non-coplanar SBRT was applied in four patients. The remaining two patients were treated with respiratory-suppressed VMAT-based SBRT; In one patient with recurrence in segment 2 (S2), the shadow of the diaphragm was used and for another patient, the shadow of lipiodol from a prior TACE treatment was used. The details of patient characteristics are shown in Table 1.

**Table 1:** Patient characteristics (N = 6).

Patient	A	B	C	D	E	F
***Tumour factors***						
Number of lesions	1	1	3	1	1	1
Size (mm)	11	120	100	65	12	36
Segment	4	6	7	2	4	6/7
serum AFP (ng/ml)	176	37275	8211	37274	5	4
Vp	3	4	3	3	3	4
***Liver functions***						
Child-Pugh score	6 A	5 A	7 B	5 A	5 A	5 A
ICG15	15.8	6.4	28.1	11.7	11.5	9.3
DCP (mAU/ml)	75	<30	31000	85	999	331
***Aetiology of liver disease***						
Hepatitis B	+	−	−	+	−	+
Hepatitis C	+	−	−	−	+	−
Alcohol abuse	−	−	+	−	−	+
BMI^$^	19.8	22.0	24.4	22.1	17.7	28.0
***Staging***						
Okuda	0–I	0–I	1–II	0–I	0–I	0–I
BCLC	C	C	C	C	C	C
CLIP	1	2	4	2	1	1

*Abbreviations:* ICG15: indocyanine green retention at 15 minutes; BMI: body mass index; AFP: alpha fetoprotein; DCP: des-γ-carboxy prothrombin; BCLC: Barcelona Clinic Liver Cancer Staging System; CLIP: Cancer of the Liver Italian Program.

^$^BMI = body mass index; body weight (kg)/height (m) ^2.

### Pathological and clinical courses

**Figure 1:**
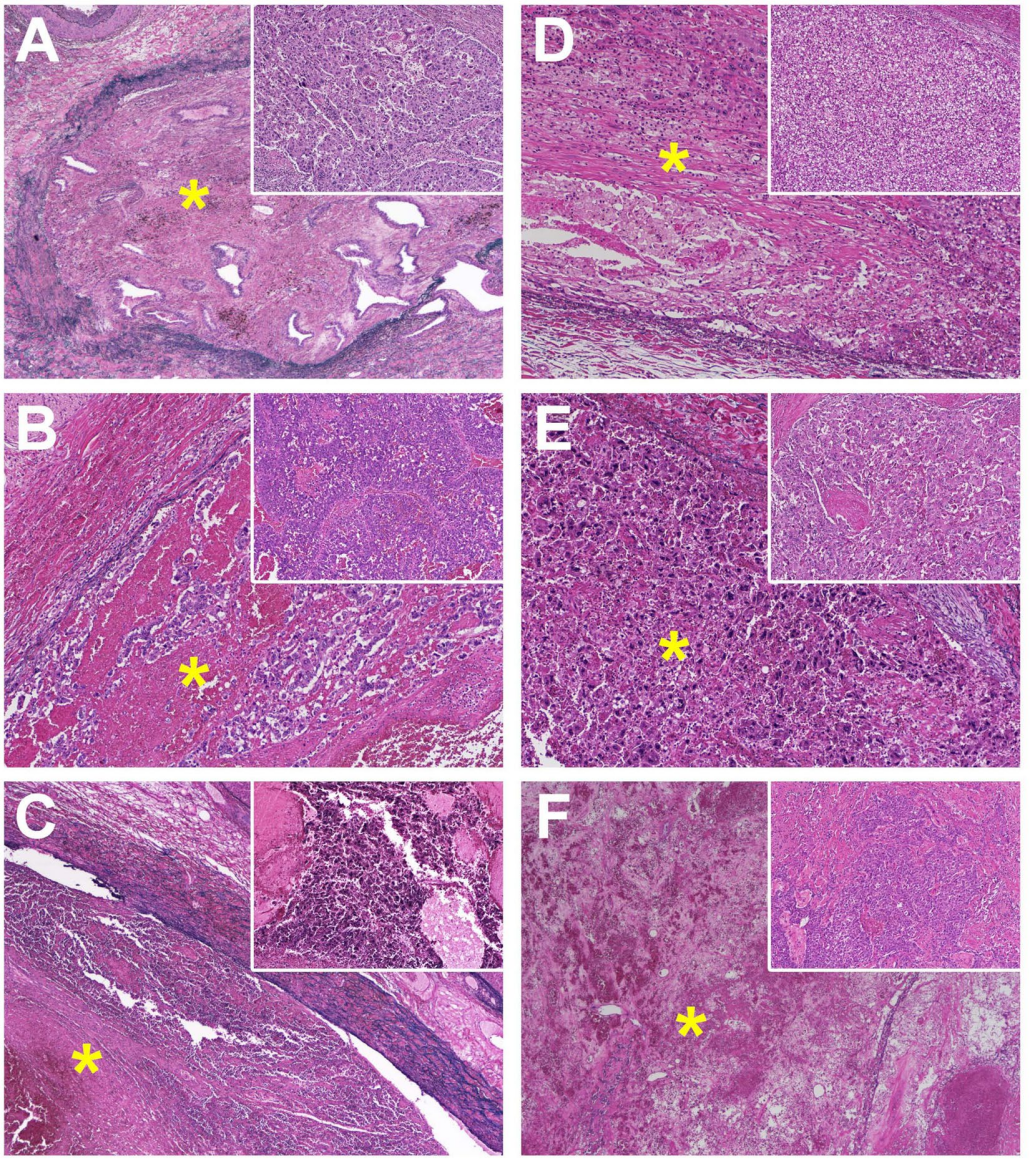
Photomicrograph of thrombi in the portal vein (*; Victoria blue and haematoxylin-eosin stain [VB-HE]) with the primary tumour features (small windows in the right upper corner; VB-HE, x 50) in hepatocellular carcinomas surgically removed in patients (**A** to **F**). Portal-vein thrombi showing no evidence of viable tumour in Patients (**A**,**C**,**F**) (VB-HE, x 40), or containing residues of tumour cells in patients (**B**,**D**,**E**) with varying degrees of degeneration (VB-HE, x 100).

The median period from the final day of SBRT to surgery was 9.5 (range, 6–17) days. All patients underwent complete surgical resection with negative margins. Pathological examination of the irradiated PVTT specimens revealed necrosis in all cases, with complete necrosis, 30% necrosis with high degeneration, and 30% necrosis without any degeneration (Patient B) were noted in 3 (50%), 2 (33%), and 1 (17%) cases, respectively. The photomicrograph of the thrombi is shown in Fig. 1. No association was noted between response to radiotherapy and tumour size, differentiation, or the period from SBRT to surgery (Table 2). Patient B experienced lung metastasis in 6.3 months and intrahepatic recurrence in 8.9 months during the follow-up period (median: 22.5 months, range: 5.9–49.6 months). The 1-year IHRFS and EHRFS were 75.0% and 83.3%, respectively.

**Table 2:** Details of radiotherapy and clinical course in hepatocellular carcinoma patients with tumour thrombus.

Patient	A	B	C	D	E	F
***SBRT technique***						
	Gat	Gat	Gat	Gat	Sup	Sup
***Dose-Volume Histogram***						
***Normal liver (Liver-PTV)***						
Volume (cc)	1070	1589	1375	1096	863	1288
Mean (Gy)	8.2	9.6	8.7	5.0	5.9	6.6
***Estimated remnant liver***						
Volume (cc)	871	365	387	970	640	995
Mean (Gy)	6.0	10.6	5.6	3.0	3.2	4.9
*PTV*						
Volume (cc)	20.0	45.0	53.9	16.9	28.1	79.8
***Dmax of organs at risk***						
Spinal cord (Gy)	4.7	10.5	16.3	2.5	4.7	12.4
Stomach (Gy)	24.8	5.1	20.7	19.3	12.3	21.5
Duodenum (Gy)	6.0	3.5	23.4	10.6	11.5	13.3
Bowel (Gy)	11.1	1.2	10.6	11.7	0.6	14.3
***Period from SBRT to operation*** (days)						
	17	6	7	10	9	12
***Pathology***						
Differentiation	P	M	W-P	W	P	P
Necrotic change	100%	30%	100%	30%	30%	100%
Degeneration		−		+	+	
***Clinical Course***						
Intrahepatic recurrence	NED	Rec	NED	NED	NED	NED
Extrahepatic recurrence	NED	Rec	NED	NED	NED	NED

*Abbreviations:* SBRT: stereotactic body radiotherapy; Gat: respiratory-gated SBRT, Sup: respiratory-suppressed SBRT, PTV: planning target volume, Dmax: maximum dose. P: Poorly differentiated, M: moderately differentiated; W: well differentiated; NED: no evidence of disease; Rec: recurrence.

### SBRT-related acute toxicity and dose-volume histogram

**Figure 2:**
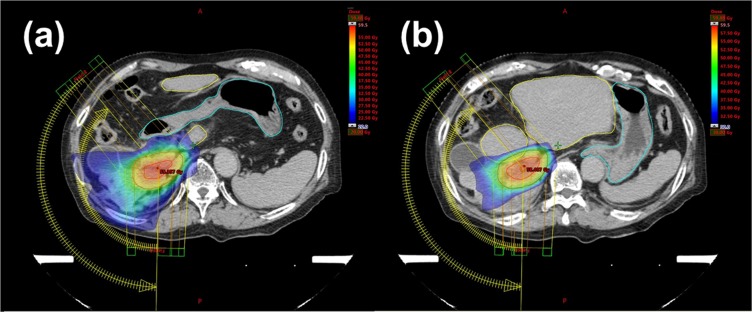
Example of a treatment plan (patient F) showing the colour wash dose distribution; (**a**) 20 Gy and above, and (**b**) 30 Gy and above. Radiation doses to the stomach and duodenum (marked by a light blue line) were less than 20 Gy; irradiation beyond the resection line (remnant liver: marked by a yellow line) was also reduced to less than 30 Gy.

Acute toxicities included grade 1 nausea and grade 1 anorexia (n = 1, each). During the follow-up period, no gastrointestinal haemorrhage, ulcers, and strictures, and classic/non-classic RILD were observed. The mean dose of the estimated remnant liver was 5.2 Gy (range: 3.0–10.7 Gy). The median values of the V20 Gy and V30 Gy of the estimated remnant liver were 33.6 cc (range: 13.2–54.1 cc) and 12.2 cc (range: 5.7–25.5 cc), respectively. The median PTV was 37.5 cc (range: 30.0–79.8 cc). Both respiratory-gated multiple non-coplanar SBRT and respiratory-suppressed VMAT-based SBRT maintained the Dmax of OARs within the tolerable range (institution dose constraints were achieved). A typical dose distribution of VMAT-based SBRT is shown in Fig. 2 (patient F).

## Discussion

This is the first report to investigate the use of preoperative respiratory-gated SBRT and respiratory-suppressed VMAT-based SBRT in the treatment of HCC with PVTT. Preoperative SBRT strategy accomplished a high resection rate and low toxicity. Conventional fractionation requires >5 weeks to deliver adequate doses for tumour control, but a long treatment time is potentially fatal to non-responders. If the surgical indication is evaluated early, non-responders can have their subsequent treatments changed from surgery to systemic therapy. Radiotherapy induces inflammation and fibroblast recruitment and leads to fibrosis in 4–12 months^22^, which may compromise the surgical procedure. Since the period from radiotherapy to surgery in our study was shorter than that of previous reports^16,17,23,24^, the patients underwent surgery before developing fibrosis (Table 3).

**Table 3:** Literature reports of preoperative radiotherapy.

Author	N	Necrosis	Preoperative Treatment	Period from RT to surgery	RR	Acute AEs*	RILD
Wada^16^	2	60–100%	3D-CRT 60 Gy / 6 fr. and 39 Gy / 13 fr.	3–4 weeks	NA	0%	0%
Kamiyama^17^	15	Viable-100%	3D-CRT 30–36 Gy / 10–12 fr.	2 weeks	NA	6.7%	0%
Choi^24^	16	30–100%	TACE + 3D-CRT 30–68 Gy	4.7 months	NA	NA	NA
Yeh^23^	12	NA	IMRT 60 Gy / 30 fr.	1.2–5 months	11.3%	0%	NA
This study	6	30–100%	SBRT (for PVTT only) 48 Gy / 4 fr.	2 weeks (6-17 days)	75%	0%	0%

*Abbreviations:* RR: resection rate; HR: hepatic resection NA: not assessed; RILD: radiation-induced liver disease; SBRT: stereotactic body radiotherapy; IMRT: intensity-modulated radiotherapy; TACE: transarterial chemoembolization; 3D-CRT: three-dimensional conformal radiotherapy; RT: radiotherapy;

*worse than grade 2.

The radiobiological mechanism of SBRT is unclear, but it is well known that high dose irradiation damages not only tumour cells but also the vascular microenvironment. The tumour and tumour blood vessel damage can cause tumour-associated antigens to be released, which triggers an anti-tumour response^25^. Reports, which focus on the post-SBRT pathology are limited. SBRT as a bridging treatment prior to liver transplantation showed a complete pathological response rate of 27–63%^26–29^. The complete pathological response rate in our study was comparable to that reported in previous studies. In non-complete pathological response patients, degeneration was observed in two of three patients. Only one patient developed minor necrosis and no degeneration. Notably, early recurrence was seen in this patient. No definite conclusions can be drawn based on this data; however, our findings indicate that the poor SBRT-response group may have a worse prognosis.

AEs are major issues in SBRT for locally advanced HCC. Bujold *et al*. and Bae *et al*. reported that SBRT-induced toxicity ≥ grade 3 was seen in 14–30% of HCCs^30,31^. In the particle radiotherapy for locally advanced HCC, the incident rate of severe toxicities was found to be low, but toxicities did occur^32–34^. The liver is a radiosensitive organ and patients with HCC frequently demonstrate background liver cirrhosis and potential liver dysfunction. In addition, as the PTV volumes positively correlate with the irradiated dose of the liver^35^, the goal in radiotherapy for locally advanced HCC is to decrease the irradiated dose to the liver and the risk of developing RILD. If the PTV is located near the gastrointestinal organs, the irradiated dose of these organs should also be lowered sufficiently to prevent haemorrhage, ulcers, and strictures. We minimized the estimated remnant liver dose using respiratory-gated SBRT and respiratory-suppressed VMAT-based SBRT, which can achieve the required target coverage, and maintain the normal tissue dose-volume constraints of other OARs within prescribed limits^36–38^. Furthermore, to decrease the irradiated volumes, we did not include the whole tumour and confined the GTV to the PVTT. As a result, only grade 1 SBRT-induced acute toxicities were observed, there were no subacute gastrointestinal toxicities and no RILD observed, though we focused on the SBRT-related toxicity in the prospective study associated with radiotherapy, surgery and chemotherapy. To evaluate the late toxicities accurately, we need to consider other interventions and further follow-up. Further data related to surgery and HAIC will be presented in a future publication.

The effectiveness of SBRT as a bridging therapy or down-staging prior to transplantation has recently been reported^39^; however, organ shortages are a global issue affecting those awaiting liver transplantation; many patients are on long-term immunosuppression after transplantation. Thus, using SBRT confined to PVTT as a down-staging treatment would be a promising option for a less invasive hepatectomy. We need to consider that a high recurrence rate has been observed in the remnant liver (intrahepatic and multifocal), as multicentric occurrence is a characteristic of HCC. Further investigation would clarify long-term outcomes of the treatment.

In patients with preserved hepatic function and with lesions confined to the hemi-lobe, SBRT may be effective as preoperative therapy in curative hepatic resection. The scope of the current study is limited by the small sample size and the short follow-up period. Further cumulative studies of patients are necessary to evaluate appropriate patient stratification, survival outcomes, and late toxicity. In addition, it should be noted that this is a study from a single institution, which is a high-volume centre for surgery in Japan. In conclusion, as a preoperative treatment, SBRT targeting PVTT in HCC were pathologically effective, and tolerable in acute phases.

## Data Availability

There are no restrictions on the availability of materials or information. The datasets generated during and/or analysed during the current study are available from the corresponding author on reasonable request.
